# Iron Intake and Associated Factors in General Japanese Population: NIPPON DATA80, NIPPON DATA90 and National Nutrition Monitoring

**DOI:** 10.2188/jea.JE20090225

**Published:** 2010-05-05

**Authors:** Tanvir Chowdhury Turin, Nagako Okuda, Katsuyuki Miura, Yasuyuki Nakamura, Nahid Rumana, Aya Kadota, Koji Tamakoshi, Hirotsugu Ueshima

**Affiliations:** 1 Department of Health Science, Shiga University of Medical Science, Ohtsu, Japan; 2 Cardiovascular Epidemiology, Kyoto Women’s University, Kyoto, Japan; 3 Department of Public Health - Health Information Dynamics, Nagoya University Graduate School of Medicine, Nagoya, Japan

**Keywords:** nutrition, diet, iron, mean intake, daily intake, density intake, population

## Abstract

**Objective:**

The purpose of this study was to investigate the dietary iron intake and associated other dietary factors and clinical characteristics among a representative sample cohort of Japanese population.

**Methods:**

We obtained data from NIPPON DATA80 and 90 that were conducted with the National Nutrition Surveys in 1980 and in 1990. Then we estimated nutrient and food intakes of individuals in the National Nutrition Survey of 1980 and that of 1990, which were adjusted on the basis of data of the National Nutrition Survey of 1995. Finally, we analyzed data for the 10 422 participants (4585 men and 5837 women) in NIPPON DATA80 and 8342 participants (3488 men and 4854 women) in NIPPON DATA90 having dietary iron intake information.

**Results:**

In NIPPON DATA80 and 90, there was a significant relationship between the dietary iron intake and age for both men and women. Dietary protein intake was associated with iron intake where as dietary fat intake did not show any association. Regarding the minerals, significant relationships were observed between the different minerals and dietary iron intake. Apart from the food group of milk and dairy products, there were significant differences in other food groups according to quintiles of iron intakes for men and women.

**Conclusions:**

We described the mean dietary iron intake and its relation with other dietary factors and clinical characteristics in Japanese adults as the baseline data in NIPPON DATA80 and in NIPPON DATA90.

## INTRODUCTION

Nutritional and lifestyle factors are key determinants of cardiovascular diseases (CVD) across populations. Accumulation of reliable data obtained from cohort studies in Japan, population with the highest longevity of the world, using representative population groups is needed to establish strategies in Japan for health promotion and prevention of lifestyle-related diseases that take into account the differences between dietary habits and prevalence of diseases in Japan and other countries. The National Nutritional Survey (NNS) was initiated in 1946 in Japan under the direction of the supreme commander of the General Headquarters with the main purpose of obtaining factual information on the nutritional health, actual food consumption and food requirements in Japan for emergency food supplies from other countries.^1^ Household-based food consumption data had been collected in order to fulfill the above purposes. Using the majority of the participants for NNS, the National Survey on Circulatory Disorders has been conducted every 10 years in order to obtain cross-sectional data on cardiovascular disease prevalence and risk factors since 1960.^2^ Two cohort studies based on the National Survey on Circulatory Disorders in 1980 and 1990^3^ have been named as the National Integrated Project for Prospective Observation of Non-communicable Disease and Its Trends in the Aged (NIPPON DATA80 and NIPPON DATA90). Data linkage was performed between NNS and NIPPON DATA with the objective to investigate fundamental data in relation between the dietary nutritional intake and the health.

To examine the intakes of iron in the Japanese population, this study analyzes the aforementioned pooled data to investigate the dietary iron intake and associated dietary factors and clinical characteristics among randomly selected representative sample cohorts of Japanese general population.

## PARTICIPANTS AND METHODS

### Nutritional survey

Food intake survey by weighed food records in three consecutive representative days were conducted by specially trained dietary interviewers. These surveys were performed for all household members from randomly selected 300 survey districts throughout Japan. Dietary interviewers visited participants’ houses at least once during the survey. Weekends and holydays were avoided. In the NNS before 1994, nutrient and food intakes per capita were calculated by dividing the amount of food intake by the number of household members. However, since 1995, nutrient and food intakes of individual household members have been calculated by proportional division method for one day, in which the amount of food intake is proportionally divided by the consumption rate of each household member. Average intakes by gender and age class could be calculated by this method. The average intakes in NNS conducted in 1995 were calculated by a combination method of household-based food weighing record and an approximation of proportions by which family members shared each dish or food in the household.

In this study, we estimated nutrient and food intakes of individuals in the National Nutrition Survey of 1980 (NNS-80) and that of 1990 (NNS-90), which were adjusted on the basis of data of the NNS of 1995. We estimated nutrient intakes of each household member by dividing household intake data of NNS-80 and -90 proportionally using average intakes by sex and age groups calculated for NNS conducted in 1995.^4^ For each person, means of the estimated individual nutrients from the three days records were used in the analyses. Detailed nutrient that were not included in the NNS were complemented via imputation method. Particulars of the nutrient intake calculation have been descried elsewhere.^5^

### NIPPON DATA cohort participants

We obtained data from NIPPON DATA80 (data from the Third National Survey on Circulatory Disorders, Japan in 1980) and NIPPON DATA90 (data from the Fourth National Survey on Circulatory Disorders, Japan in 1990), that were conducted with the National Nutrition Surveys in 1980 and in 1990. NIPPON DATA80 and 90 have been described in detail in previously.^6^^–^^9^ These surveys were performed for all household members aged 30 years or older in 300 census tracts, which were selected using the stratified random sampling method, based on the national census of 1975, throughout Japan. The survey consisted of physical examinations, serological examination, and a self-administered questionnaire on lifestyle, including an essential nutrition survey using the food-frequency method. Trained staff at local health centers in the respective districts performed the examinations in community centers. At baseline, body height and body weight was measured under light clothes and body mass index (BMI) was calculated by body weight (kg) divided by square of body height (m). Baseline blood pressures were measured by trained public health nurses of each public health centre on the right arm of participants by using a standard mercury sphygmomanometer while the participants were seated and after they had rested for more than 5 minutes.

### Data analyses

A total of 10 422 participants (men: 4585 & women: 5837) of NIPPON DATA80 and 8342 participants (men: 3488 & women: 4854) with dietary iron intake information were included in the present study. Data were analyzed in men and women separately. Systolic blood pressure (SBP), diastolic blood pressure (DBP), BMI, fatty acids intakes, mineral and food group intakes were examined by quintiles of dietary iron intake. Data are presented as means and standard deviations. Chi-squared tests were used for the categorical variables. To detect differences in continuous variables in groups, analysis of variance (ANOVA) was used. The “contrast” option for analysis of variance was used to detect deviation from linearity in the association between continuous variables and the five iron intake groups, and trend *P* was obtained. All statistical analyses were performed using SAS® version 9.1 (SAS Institute, Cary, NC.).

## RESULTS

For the participants of NIPPON DATA80 the mean dietary iron intake for men and women was 15.0 (SD 3.94) mg/day and 13.5 (SD 3.55) mg/day, respectively. The mean dietary iron density intake was 6.30 (SD 1.19) mg/1000 kcal for men and 7.02 (SD 1.32) mg/1000 kcal for women. From the data obtained from NIPPON DATA90, the estimated mean dietary iron intake for men and women was 12.7 (SD 3.44) mg/day and 11.2 (SD 3.03) mg/day, respectively. The mean dietary iron density intake was 5.54 (SD 1.17) mg/1000 kcal for men and 6.04 (SD 1.25) mg/1000 kcal for women.

NIPPON DATA80 participant level variables (A) and nutrient intakes (B) according to quintiles of dietary iron intake for men are shown in Table 1 and variables according to quintiles of dietary iron intake for women are shown in Table 2. Higher iron intake was associated with higher age, systolic and diastolic BP, protein intake, minerals (including potassium, calcium, sodium), vitamins (including Vitamins A, B1, B2, C), and fiber intake for both men and women. On the other hand, BMI, total dietary cholesterol did not reveal any association with the dietary iron for men.

**Table 1.** Participant characteristics (A) and nutrient intake (B) according to quintiles of dietary iron intake for men: NIPPON DATA80

	Iron intake quintiles (mg/1000 kcal)											
	
Range	3.0–5.3		5.4–5.9		6.0–6.4		6.5–7.1		7.2–14.3		*P* diff	*P* trend
	
	Mean	*SD*	Mean	*SD*	Mean	*SD*	Mean	*SD*	Mean	*SD*		
*n*	905		1013		833		902		932			
**(A)**												
Age (year)	46.2	*12.1*	47.9	*12.7*	49.6	*13.6*	51.8	*13.3*	54.4	*13.6*	<0.001	<0.001
BMI (kg/m^2^)	22.4	*2.8*	22.4	*2.9*	22.6	*2.8*	22.5	*3.0*	22.7	*2.9*	0.075	0.220
SBP (mm Hg)	135.0	*20.8*	136.5	*20.6*	138.7	*21.2*	139.7	*21.9*	142.0	*20.7*	<0.001	<0.001
DBP (mm Hg)	82.1	*12.4*	83.2	*12.0*	83.7	*12.1*	84.1	*12.9*	84.5	*12.7*	<0.001	0.003
Current smoking (%)	66.2		63.4		64.2		60.4		60.9		0.066	
Current drinking (%)	76.5		74.9		75.4		73.1		71.5		0.108	
**(B)**												
Total energy (kcal)	2461.6	*546.3*	2439.9	*470.7*	2419.0	*476.1*	2386.2	*476.4*	2299.4	*522.5*	<0.001	0.001
Carbohydrate (%kcal)	60.9	*6.4*	60.1	*6.0*	59.9	*6.1*	59.2	*6.3*	58.7	*7.1*	<0.001	<0.001
Protein (%kcal)	13.4	*1.5*	14.4	*1.4*	14.9	*1.6*	15.6	*1.7*	17.0	*2.3*	0.378	0.123
Total fat (%kcal)	19.7	*5.7*	20.1	*5.1*	20.1	*5.0*	20.1	*4.9*	19.9	*5.1*	<0.001	<0.001
SFA (%kcal)	5.6	*1.6*	5.7	*1.4*	5.7	*1.4*	5.7	*1.4*	5.6	*1.5*	0.096	0.891
MUFA (%kcal)	7.5	*2.1*	7.5	*1.9*	7.4	*1.9*	7.5	*1.9*	7.3	*2.0*	<0.001	<0.001
PUFA (%kcal)	5.1	*1.5*	5.2	*1.4*	5.2	*1.3*	5.4	*1.4*	5.4	*1.4*	<0.001	<0.001
Potassium (mg/1000 kcal)	1056.5	*159.0*	1182.2	*156.2*	1269.1	*167.7*	1348.0	*175.7*	1519.9	*236.5*	<0.001	<0.001
Calcium (mg/1000 kcal)	176.9	*44.6*	207.8	*39.3*	226.7	*42.7*	247.6	*45.2*	288.4	*61.4*	<0.001	<0.001
Sodium (mg/1000 kcal)	1837.8	*500.0*	2231.5	*505.5*	2442.0	*547.7*	2684.9	*672.6*	3260.3	*1012.2*	<0.001	<0.001
Vitamin A (IU/1000 kcal)	534.7	*196.1*	649.4	*220.5*	721.8	*269.0*	790.5	*292.8*	938.9	*403.7*	<0.001	<0.001
Vitamin B1 (mg/1000 kcal)	0.4	*0.2*	0.5	*0.2*	0.5	*0.2*	0.5	*0.2*	0.5	*0.2*	<0.001	<0.001
Vitamin B2 (mg/1000 kcal)	0.5	*0.1*	0.5	*0.1*	0.5	*0.1*	0.6	*0.1*	0.6	*0.2*	<0.001	<0.001
Vitamin C (mg/1000 kcal)	35.1	*12.9*	41.7	*13.8*	46.4	*15.0*	50.4	*16.8*	58.2	*22.2*	<0.001	<0.001

*P* diff values obtained by ANOVA or Chi square statistics. *P* trend obtained by contrast statement of analysis of variance.

BMI, body mass index; SBP, systolic Blood Pressure; DBP, diastolic blood pressure; SFA, saturated fatty acid; MUFA, mono unsaturated fatty acid; PUFA, poly unsaturated fatty acid.

**Table 2.** Participant characteristics (A) and nutrient intake (B) according to quintiles of dietary iron intake for women: NIPPON DATA80

	Iron intake quintiles (mg/1000 kcal)											
	
Range	3.2–5.9		6.0–6.5		6.6–7.1		7.2–8.0		8.1–15.5		*P* diff	*P* trend
	
	Mean	*SD*	Mean	*SD*	Mean	*SD*	Mean	*SD*	Mean	*SD*		
*n*	1174		1146		1117		1262		1138			
**(A)**												
Age (year)	46.2	*13.2*	47.3	*13.2*	49.6	*13.2*	51.9	*13.2*	55.2	*12.6*	<0.001	<0.001
BMI (kg/m^2^)	22.6	*3.4*	22.4	*3.0*	22.9	*3.5*	23.0	*3.5*	23.2	*3.3*	<0.001	<0.001
SBP (mm Hg)	130.5	*21.8*	131.5	*21.1*	133.6	*22.3*	135.2	*21.5*	138.1	*22.4*	<0.001	<0.001
DBP (mm Hg)	78.1	*12.7*	78.5	*11.7*	79.5	*11.9*	80.0	*11.8*	81.4	*12.3*	<0.001	<0.001
Current smoking (%)	11.9		8.5		9.4		9.7		9.6		0.072	
Current drinking (%)	22.6		23.0		19.7		18.6		17.8		0.004	
**(B)**												
Total energy (kcal)	1987.3	*428.7*	1956.5	*373.5*	1944.5	*371.2*	1910.8	*389.5*	1848.2	*431.5*	<0.001	<0.001
Carbohydrate (%kcal)	63.3	*6.8*	62.1	*6.4*	62.2	*6.6*	61.7	*6.8*	61.1	*7.4*	<0.001	<0.001
Protein (%kcal)	13.8	*1.4*	14.7	*1.5*	15.3	*1.6*	16.0	*1.8*	17.5	*2.2*	<0.001	<0.001
Total fat (%kcal)	21.3	*6.1*	22.1	*5.6*	21.8	*5.7*	21.9	*5.6*	21.4	*5.9*	0.002	0.056
SFA (%kcal)	6.2	*1.8*	6.3	*1.6*	6.2	*1.6*	6.2	*1.6*	6.0	*1.7*	<0.001	0.958
MUFA (%kcal)	8.1	*2.3*	8.3	*2.1*	8.2	*2.1*	8.2	*2.1*	7.9	*2.3*	<0.001	0.663
PUFA (%kcal)	5.5	*1.5*	5.8	*1.5*	5.7	*1.5*	5.86	*1.6*	5.9	*1.5*	<0.001	<0.001
Potassium (mg/1000 kcal)	1202.1	*179.4*	1326.5	*182.7*	1417.9	*188.9*	1524.0	*204.6*	1742.6	*279.3*	<0.001	<0.001
Calcium (mg/1000 kcal)	217.3	*49.6*	247.9	*46.4*	270.1	*51.2*	295.6	*51.6*	348.4	*71.6*	<0.001	<0.001
Sodium (mg/1000 kcal)	1991.4	*516.9*	2375.3	*528.4*	2591.2	*577.4*	2889.3	*745.7*	3472.4	*1022.5*	<0.001	<0.001
Vitamin A (IU/1000 kcal)	637.8	*228.3*	751.4	*264.9*	823.0	*298.1*	929.9	*349.5*	1140.8	*497.4*	<0.001	<0.001
Vitamin B1 (mg/1000 kcal)	0.6	*0.2*	0.6	*0.2*	0.6	*0.2*	0.6	*0.2*	0.6	*0.2*	<0.001	<0.001
Vitamin B2 (mg/1000 kcal)	0.3	*0.1*	0.4	*0.1*	0.4	*0.1*	0.4	*0.1*	0.5	*0.1*	<0.001	<0.001
Vitamin C (mg/1000 kcal)	46.8	*16.7*	54.7	*18.5*	59.6	*18.8*	66.3	*22.1*	79.0	*29.3*	<0.001	<0.001

*P* diff' values obtained by ANOVA or Chi square statistics. *P* trend obtained by contrast statement of analysis of variance.

BMI, body mass index; SBP, systolic Blood Pressure; DBP, diastolic blood pressure; SFA, saturated fatty acid; MUFA, mono unsaturated fatty acid; PUFA, poly unsaturated fatty acid.

Table 3 and 4 shows the food group intakes according to quintiles of dietary iron intake for men and women of NIPPON DATA80, respectively. Regarding food groups, lower amount of dietary cereals, rice, flour, fats and oils were associated with higher dietary iron for both men and women. On the other hand, higher intake of nuts, potatoes, sugar and sweets, soy beans, fruits, vegetables, mushrooms, sea algae, fish and shellfish, egg were associated with higher dietary iron.

**Table 3.** Nutrient intakes of different food group according to quintiles of dietary iron intake for men: NIPPON DATA80

	Iron intake quintiles (mg/1000 kcal)											
	
Range	3.0–5.3		5.4–5.9		6.0–6.4		6.5–7.1		7.2–14.3		*P* diff	*P* trend
	
	Mean	*SD*	Mean	*SD*	Mean	*SD*	Mean	*SD*	Mean	*SD*		
*n*	905		1013		833		902		932			
Cereals (g/1000 kcal)	174.9	*29.2*	164.0	*28.0*	160.3	*28.5*	154.6	*29.2*	146.9	*30.9*	<0.001	<0.001
Rice (g/1000 kcal)	140.0	*36.4*	129.9	*31.9*	126.5	*32.9*	120.8	*32.6*	114.7	*34.0*	<0.001	<0.001
Flour product (g/1000 kcal)	38.3	*27.0*	37.2	*24.6*	36.8	*24.0*	35.8	*24.2*	34.0	*25.7*	0.004	0.043
Nuts (g/1000 kcal)	0.4	*1.5*	0.5	*1.7*	0.6	*1.9*	0.6	*1.7*	0.7	*1.8*	0.009	0.009
Potatoes (g/1000 kcal)	21.6	*17.6*	26.4	*17.4*	28.1	*18.1*	30.2	*19.9*	32.2	*21.4*	<0.001	<0.001
Sugar & sweetener (g/1000 kcal)	5.0	*3.6*	5.8	*4.3*	5.7	*4.0*	5.9	*4.1*	6.0	*4.4*	<0.001	<0.001
Sweet & snacks (g/1000 kcal)	5.0	*6.2*	6.3	*6.9*	6.6	*7.3*	6.5	*6.9*	7.2	*8.3*	<0.001	<0.001
Fats & Oils (g/1000 kcal)	8.1	*5.0*	7.6	*4.4*	7.1	*4.1*	6.8	*4.0*	5.8	*3.8*	<0.001	<0.001
Soy beans and product (g/1000 kcal)	21.8	*14.4*	29.9	*15.7*	34.2	*17.8*	39.5	*20.1*	48.3	*25.8*	<0.001	<0.001
Fruit (g/1000 kcal)	46.6	*36.0*	53.6	*33.1*	61.0	*37.7*	64.0	*40.1*	70.5	*46.8*	<0.001	<0.001
Green & yellow vegetable (g/1000 kcal)	15.9	*10.2*	20.4	*12.1*	23.1	*14.2*	26.8	*16.2*	32.9	*22.1*	<0.001	<0.001
Other vegetable (g/1000 kcal)	76.8	*27.1*	87.4	*30.5*	96.0	*32.7*	103.5	*37.1*	120.0	*50.2*	<0.001	<0.001
Mushrooms (g/1000 kcal)	3.2	*4.2*	3.7	*5.0*	4.2	*5.3*	4.5	*5.8*	5.4	*6.6*	<0.001	<0.001
Sea algae (g/1000 kcal)	1.3	*1.2*	1.8	*1.8*	2.4	*2.4*	3.0	*3.1*	5.0	*5.6*	<0.001	<0.001
Condiment & beverage (g/1000 kcal)	81.9	*94.9*	78.0	*65.5*	80.6	*64.5*	83.2	*74.1*	87.9	*73.8*	0.059	0.566
Fish & shellfish (g/1000 kcal)	41.1	*19.9*	47.1	*21.5*	51.4	*22.2*	56.3	*24.8*	65.8	*30.5*	<0.001	<0.001
Meat (g/1000 kcal)	31.1	*16.3*	30.4	*15.4*	29.4	*15.6*	28.6	*15.3*	27.9	*19.4*	<0.001	0.001
Egg (g/1000 kcal)	14.7	*8.7*	16.4	*7.9*	17.4	*8.8*	18.0	*9.6*	19.4	*12.0*	<0.001	<0.001
Milk & dairy (g/1000 kcal)	28.8	*25.5*	30.6	*22.6*	31.2	*25.3*	31.8	*24.9*	29.3	*26.8*	0.065	0.012
Other food (g/1000 kcal)	2.4	*6.0*	2.6	*4.9*	2.9	*6.0*	3.0	*6.4*	3.1	*10.3*	0.169	0.058

*P* diff values obtained by ANOVA statistics. *P* trend obtained by contrast statement of analysis of variance.

**Table 4.** Nutrient intakes of different food group according to quintiles of dietary iron intake for women: NIPPON DATA80

	Iron intake quintiles (mg/1000 kcal)											
	
Range	3.2–5.9		6.0–6.5		6.6–7.1		7.2–8.0		8.1–15.5		*P* diff	*P* trend
	
	Mean	*SD*	Mean	*SD*	Mean	*SD*	Mean	*SD*	Mean	*SD*		
*n*	1174		1146		1117		1262		1138			
Cereals (g/1000 kcal)	168.1	*27.9*	158.4	*27.7*	154.9	*27.5*	149.2	*28.6*	141.9	*30.3*	<0.001	<0.001
Rice (g/1000 kcal)	119.4	*34.1*	110.3	*31.2*	109.0	*32.1*	105.5	*31.7*	101.6	*32.5*	<0.001	<0.001
Flour product (g/1000 kcal)	46.4	*29.0*	46.5	*29.7*	44.5	*28.1*	42.6	*28.1*	39.1	*29.4*	<0.001	<0.001
Nuts (g/1000 kcal)	0.7	*2.7*	0.7	*2.3*	0.9	*2.9*	0.8	*2.1*	1.1	*2.7*	0.001	0.221
Potatoes (g/1000 kcal)	27.3	*19.2*	31.7	*21.2*	32.9	*21.3*	36.4	*24.0*	39.2	*28.8*	<0.001	<0.001
Sugar & sweetener (g/1000 kcal)	6.4	*4.5*	7.0	*4.8*	7.0	*5.0*	6.9	*4.7*	6.7	*4.9*	0.010	0.008
Sweet & snacks (g/1000 kcal)	12.2	*13.2*	13.3	*12.9*	13.9	*13.5*	14.2	*14.4*	13.0	*14.3*	0.003	<0.001
Fats & Oils (g/1000 kcal)	8.9	*5.3*	8.8	*4.9*	8.0	*4.4*	7.6	*4.5*	6.6	*4.3*	<0.001	<0.001
Soy beans and product (g/1000 kcal)	24.2	*15.3*	31.6	*16.8*	35.6	*18.6*	43.1	*22.0*	52.2	*27.4*	<0.001	<0.001
Fruit (g/1000 kcal)	79.3	*53.3*	89.1	*52.5*	95.9	*56.3*	101.4	*59.4*	114.7	*72.4*	<0.001	<0.001
Green & yellow vegetable (g/1000 kcal)	20.5	*13.1*	25.4	*15.1*	29.1	*17.7*	34.3	*20.7*	44.0	*28.9*	<0.001	<0.001
Other vegetable (g/1000 kcal)	86.9	*32.3*	99.5	*34.4*	108.1	*37.0*	117.2	*43.2*	135.9	*55.1*	<0.001	<0.001
Mushrooms (g/1000 kcal)	3.4	*4.6*	4.1	*5.3*	4.6	*5.9*	5.1	*6.4*	6.2	*7.5*	<0.001	<0.001
Sea algae (g/1000 kcal)	1.4	*1.5*	2.0	*2.0*	2.7	*2.9*	3.7	*3.7*	6.1	*6.9*	<0.001	<0.001
Condiment & beverage (g/1000 kcal)	34.9	*51.0*	33.5	*31.0*	33.7	*28.8*	35.2	*32.3*	38.4	*52.3*	0.025	0.809
Fish & shellfish (g/1000 kcal)	39.7	*19.2*	44.9	*20.5*	48.9	*21.0*	53.7	*24.3*	64.4	*29.1*	<0.001	<0.001
Meat (g/1000 kcal)	28.4	*15.0*	28.9	*15.2*	28.4	*14.6*	27.4	*15.1*	26.5	*19.3*	0.002	0.100
Egg (g/1000 kcal)	16.1	*9.1*	17.6	*8.6*	18.3	*9.5*	19.3	*10.2*	20.6	*13.1*	<0.001	<0.001
Milk & dairy (g/1000 kcal)	48.4	*38.0*	49.0	*33.6*	49.2	*36.7*	46.2	*35.6*	44.3	*39.8*	0.005	0.176
Other food (g/1000 kcal)	2.5	*6.0*	3.1	*6.4*	3.3	*6.5*	3.4	*7.2*	3.5	*8.7*	0.003	0.001

*P* diff values obtained by ANOVA statistics. *P* trend obtained by contrast statement of analysis of variance.

Table 5 shows participant level variables (A) and nutrient intakes (B) according to quintiles of dietary iron intake for men for NIPPON DATA90. Table 6 shows participant level variables (A) and nutrient variables (B) according to quintiles of dietary iron intake for women. Higher iron intake was associated with higher age, systolic and diastolic BP, protein intake, fiber, minerals (including potassium, calcium, sodium, phosphorus, magnesium), vitamins (including Vitamins A, B1, B2, C, Niacin, D and E), and fiber intake for both men and women. On the other hand, lower carbohydrate intake and total energy were associated with higher dietary iron for both men and women. Intake of total fat, saturated fatty acid (SFA), mono-unsaturated fatty acid (MUFA), did not reveal any association with the dietary iron. Table 7 and Table 8 show the food groups according to quintiles of dietary iron intake for men and women of NIPPON DATA90 cohort, respectively. Regarding food groups, lower amount of dietary cereals, rice, flour, fats and oils were associated with higher dietary iron for both men and women. On the other hand, higher intake of nuts, potatoes, soy beans, fruits, vegetables, mushrooms, sea algae, fish and shellfish, egg were associated with higher dietary iron. Food groups of sugar and sweetener, meat did not show any association with dietary iron.

**Table 5.** Participant characteristics (A) and nutrient intake (B) according to quintiles of dietary iron intake for men: NIPPON DATA90

	Iron intake quintiles (mg/1000 kcal)											
	
Range	2.7–4.7		4.7–5.2		5.2–5.6		5.6–6.3		6.3–16.0		*P* diff	*P* trend
	
	Mean	*SD*	Mean	*SD*	Mean	*SD*	Mean	*SD*	Mean	*SD*		
*n*	697		697		699		699		696			
**(A)**												
Age (year)	48.7	*13.4*	51.4	*13.0*	53.0	*13.8*	55.8	*13.7*	57.6	*12.9*	<0.001	<0.001
BMI (kg/m^2^)	22.7	*2.9*	23.2	*3.0*	22.8	*2.9*	22.8	*3.1*	23.2	*3.1*	0.002	0.794
SBP (mm Hg)	135.2	*19.9*	136.0	*19.2*	136.6	*20.2*	139.4	*19.4*	141.1	*20.7*	<0.001	<0.001
DBP (mm Hg)	82.5	*11.1*	83.3	*11.5*	83.1	*12.2*	84.1	*11.4*	84.8	*11.8*	0.002	0.014
Current smoking (%)	62.7		57.1		55.4		48.9		48.9		<0.001	
Current drinking (%)	59.3		59.5		57.4		55.5		54.5		0.220	
**(B)**												
Total energy (kcal)	2414.1	*493.1*	2376.0	*442.4*	2317.9	*438.8*	2251.0	*450.5*	2220.7	*452.5*	<0.001	<0.001
Total carbohydrate (%kcal)	57.6	*5.9*	56.9	*5.5*	56.5	*5.5*	56.4	*6.0*	56.2	*6.0*	<0.001	<0.001
Dietary fiber (g/1000 kcal)	5.6	*1.3*	6.4	*1.4*	7.0	*1.5*	7.5	*1.7*	8.7	*2.3*	<0.001	<0.001
Protein (%kcal)	14.0	*1.6*	15.0	*1.4*	15.6	*1.6*	16.2	*1.6*	17.1	*2.0*	<0.001	<0.001
Animal protein (%kcal)	8.5	*2.1*	9.1	*1.9*	9.5	*2.1*	9.8	*2.2*	10.2	*2.4*	<0.001	<0.001
Vegetable protein (%kcal)	6.6	*0.8*	6.9	*0.6*	7.1	*0.8*	7.3	*0.9*	7.8	*1.1*	<0.001	<0.001
Total fat (%kcal)	21.9	*4.8*	22.3	*4.3*	22.5	*4.2*	22.4	*4.6*	22.4	*4.4*	0.199	0.054
Animal fat (%kcal)	10.5	*3.3*	10.7	*3.0*	11.1	*3.2*	10.9	*3.3*	11.1	*3.4*	<0.001	0.001
Vegetable fat (%kcal)	11.5	*3.7*	11.7	*3.1*	11.4	*3.0*	11.5	*3.1*	11.3	*3.1*	0.350	0.508
Dietary cholesterol (mg/1000 kcal)	165.7	*48.8*	178.9	*49.8*	187.0	*50.3*	189.2	*52.8*	198.6	*58.9*	<0.001	<0.001
SFA (%kcal)	5.8	*1.5*	5.9	*1.3*	5.99	*1.3*	5.9	*1.4*	6.0	*1.4*	0.078	0.019
MUFA (%kcal)	7.8	*1.9*	8.0	*1.7*	8.0	*1.6*	7.9	*1.8*	7.9	*1.8*	0.644	0.383
PUFA (%kcal)	5.4	*1.4*	5.5	*1.2*	5.5	*1.2*	5.6	*1.3*	5.8	*1.4*	<0.001	<0.001
Potassium (mg/1000 kcal)	1075.4	*165.9*	1218.6	*173.3*	1315.6	*187.2*	1407.6	*213.0*	1565.2	*267.6*	<0.001	<0.001
Calcium (mg/1000 kcal)	190.6	*53.8*	215.7	*52.7*	236.2	*55.4*	258.4	*62.8*	303.0	*83.4*	<0.001	<0.001
Magnesium (mg/1000 kcal)	113.6	*20.4*	128.1	*23.9*	135.7	*25.3*	142.4	*24.6*	157.0	*33.1*	<0.001	<0.001
Sodium (mg/1000 kcal)	2142.0	*527.6*	2388.3	*565.8*	2488.9	*557.5*	2671.2	*753.9*	2841.4	*809.0*	<0.001	<0.001
Phosphorus (mg/1000 kcal)	520.1	*53.7*	554.1	*49.7*	578.0	*54.0*	601.5	*56.0*	640.9	*68.5*	<0.001	<0.001
Vitamin A (IU/1000 kcal)	840.4	*510.2*	977.5	*572.1*	1183.3	*794.4*	1369.5	*1025.0*	1903.8	*2098.7*	<0.001	<0.001
Vitamin B1 (mg/1000 kcal)	0.5	*0.2*	0.6	*0.2*	0.6	*0.2*	0.6	*0.2*	0.7	*0.2*	<0.001	<0.001
Vitamin B2 (mg/1000 kcal)	0.5	*0.2*	0.6	*0.1*	0.6	*0.1*	0.7	*0.1*	0.7	*0.2*	<0.001	<0.001
Niacin (mg/1000 kcal)	7.7	*1.5*	8.2	*1.4*	8.5	*1.6*	8.8	*1.7*	9.4	*2.0*	<0.001	<0.001
Vitamin C (mg/1000 kcal)	44.2	*26.3*	50.0	*19.7*	55.3	*21.3*	59.5	*23.3*	67.5	*32.5*	<0.001	<0.001
Vitamin D (mg/1000 kcal)	41.05	*36.61*	56.79	*52.85*	55.81	*54.81*	61.16	*54.20*	72.86	*65.32*	<0.001	<0.001
Vitamin E (mg/1000 kcal)	3.9	*0.8*	4.2	*0.8*	4.2	*0.8*	4.4	*0.8*	4.7	*1.1*	<0.001	<0.001

*P* diff values obtained by ANOVA or Chi square statistics. *P* trend obtained by contrast statement of analysis of variance.

BMI, body mass index; SBP, systolic Blood Pressure; DBP, diastolic blood pressure; SFA, saturated fatty acid; MUFA, mono unsaturated fatty acid; PUFA, poly unsaturated fatty acid.

**Table 6.** Participant characteristics (A) and nutrient intake (B) according to quintiles of dietary iron intake for women: NIPPON DATA90

	Iron intake quintiles (mg/1000 kcal)											
	
Range	2.0–5.1		5.1–5.6		5.6–6.2		6.2–6.9		6.9–17.5		*P* diff	*P* trend
	
	Mean	*SD*	Mean	*SD*	Mean	*SD*	Mean	*SD*	Mean	*SD*		
*n*	970		971		970		971		972			
**(A)**												
Age (year)	47.3	*14.1*	50.5	*13.8*	53.0	*14.2*	55.3	*13.3*	58.0	*12.7*	<0.001	<0.001
BMI (kg/m^2^)	22.5	*3.3*	22.8	*3.2*	22.9	*3.5*	22.9	*3.2*	23.0	*3.3*	0.001	0.002
SBP (mm Hg)	129.3	*21.2*	132.0	*20.3*	134.2	*21.0*	135.2	*20.5*	137.7	*20.5*	<0.001	<0.001
DBP (mm Hg)	78.2	*12.6*	78.7	*11.3*	79.7	*11.6*	80.0	*11.5*	80.9	*11.8*	<0.001	<0.001
Current smoking (%)	17.7		9.5		8.9		7.8		7.1		<0.001	
Current drinking (%)	7.8		6.8		6.6		5.6		5.6		0.210	
**(B)**												
Total energy (kcal)	1949.0	*372.8*	1896.2	*360.8*	1836.7	*343.1*	1825.0	*370.5*	1787.0	*363.8*	<0.001	<0.001
Total carbohydrate (%kcal)	59.2	*6.5*	59.0	*6.1*	59.0	*5.9*	58.6	*6.0*	58.7	*6.2*	0.172	0.036
Dietary fiber (g/1000 kcal)	6.7	*1.5*	7.6	*1.6*	8.3	*1.7*	8.9	*1.9*	10.4	*2.6*	<0.001	<0.001
Protein (%kcal)	14.3	*1.6*	15.3	*1.5*	16.0	*1.6*	16.6	*1.7*	17.6	*2.0*	<0.001	<0.001
Animal protein (%kcal)	8.7	*2.0*	9.4	*2.1*	9.7	*2.1*	10.1	*2.2*	10.5	*2.5*	<0.001	<0.001
Vegetable protein (%kcal)	6.7	*0.8*	7.0	*0.8*	7.3	*0.8*	7.5	*0.9*	7.9	*1.1*	<0.001	<0.001
Total fat (%kcal)	24.6	*5.5*	24.5	*5.1*	24.4	*4.8*	24.4	*4.9*	24.2	*4.9*	0.558	0.431
Animal fat (%kcal)	11.1	*3.5*	11.4	*3.4*	11.4	*3.5*	11.5	*3.4*	11.5	*3.6*	0.076	0.013
Vegetable fat (%kcal)	13.5	*4.1*	13.1	*3.7*	13.0	*3.5*	12.9	*3.6*	12.7	*3.4*	<0.001	<0.001
Dietary cholesterol (mg/1000 kcal)	182.1	*54.0*	196.5	*57.0*	200.4	*56.7*	206.8	*58.7*	214.7	*64.1*	<0.001	<0.001
SFA (%kcal)	6.6	*1.7*	6.5	*1.5*	6.5	*1.5*	6.5	*1.5*	6.4	*1.5*	0.285	0.163
MUFA (%kcal)	8.8	*2.2*	8.7	*1.9*	8.7	*1.9*	8.6	*2.0*	8.5	*1.9*	0.079	0.077
PUFA (%kcal)	6.0	*1.5*	6.00	*1.3*	6.0	*1.3*	6.1	*1.4*	6.2	*1.4*	<0.001	0.005
Potassium (mg/1000 kcal)	1218.4	*186.2*	1367.3	*198.1*	1466.9	*205.3*	1587.4	*249.9*	1789.8	*302.6*	<0.001	<0.001
Calcium (mg/1000 kcal)	229.6	*59.2*	256.8	*63.0*	277.5	*62.9*	305.0	*74.7*	358.0	*96.8*	<0.001	<0.001
Magnesium (mg/1000 kcal)	121.5	*21.5*	135.4	*24.1*	143.0	*26.3*	151.0	*28.2*	168.7	*36.9*	<0.001	<0.001
Sodium (mg/1000 kcal)	2344.5	*581.3*	2564.7	*608.4*	2749.0	*758.5*	2884.2	*776.7*	3075.3	*894.0*	<0.001	<0.001
Phosphorus (mg/1000 kcal)	541.4	*55.2*	577.5	*55.7*	601.4	*55.4*	627.0	*60.2*	669.9	*72.8*	<0.001	<0.001
Vitamin A (IU/1000 kcal)	951.4	*515.6*	1153.1	*722.0*	1322.6	*854.8*	1553.5	*1043.4*	2191.9	*2424.1*	<0.001	<0.001
Vitamin B1 (mg/1000 kcal)	0.6	*0.2*	0.6	*0.2*	0.6	*0.2*	0.6	*0.2*	0.7	*0.2*	<0.001	<0.001
Vitamin B2 (mg/1000 kcal)	0.6	*0.1*	0.6	*0.1*	0.7	*0.1*	0.7	*0.1*	0.8	*0.2*	<0.001	<0.001
Niacin (mg/1000 kcal)	7.4	*1.4*	7.9	*1.4*	8.2	*1.4*	8.5	*1.6*	9.2	*1.9*	<0.001	<0.001
Vitamin C (mg/1000 kcal)	56.3	*28.5*	63.1	*24.5*	70.8	*26.5*	76.1	*29.3*	90.0	*40.0*	<0.001	<0.001
Vitamin D (mg/1000 kcal)	43.9	*39.8*	56.9	*51.8*	59.5	*56.1*	64.6	*56.5*	73.0	*61.0*	<0.001	<0.001
Vitamin E (mg/1000 kcal)	4.4	*0.9*	4.6	*0.9*	4.8	*0.9*	5.0	*1.0*	5.4	*1.3*	<0.001	<0.001

*P* diff values obtained by ANOVA or Chi square statistics. *P* trend obtained by contrast statement of analysis of variance.

BMI, body mass index; SBP, systolic Blood Pressure; DBP, diastolic blood pressure; SFA, saturated fatty acid; MUFA, mono unsaturated fatty acid; PUFA, poly unsaturated fatty acid.

**Table 7.** Nutrient intakes of different food group according to quintiles of dietary iron intake for men: NIPPON DATA90

	Iron intake quintiles (mg/1000 kcal)											
	
Range	2.7–4.7		4.7–5.2		5.2–5.6		5.6–6.3		6.3–16.0		*P* diff	*P* trend
	
	Mean	*SD*	Mean	*SD*	Mean	*SD*	Mean	*SD*	Mean	*SD*		
*n*	697		697		699		699		696			
Cereals (g/1000 kcal)	149.5	*27.4*	144.4	*27.0*	142.3	*25.7*	138.1	*31.0*	133.5	*28.7*	<0.001	<0.001
Rice (g/1000 kcal)	100.6	*30.4*	99.8	*29.3*	98.5	*28.3*	97.7	*31.7*	93.2	*28.5*	<0.001	<0.001
Flour product (g/1000 kcal)	46.7	*27.8*	42.9	*26.2*	42.3	*28.0*	39.2	*25.3*	38.3	*27.1*	0.003	0.014
Nuts (g/1000 kcal)	0.6	*1.9*	0.7	*2.1*	0.8	*2.3*	0.9	*2.2*	1.1	*2.4*	<0.001	<0.001
Potatoes (g/1000 kcal)	29.4	*19.6*	33.5	*21.5*	35.5	*21.3*	38.4	*23.4*	41.4	*24.7*	<0.001	<0.001
Sugar & sweetener (g/1000 kcal)	5.9	*4.2*	6.3	*5.0*	6.4	*4.8*	6.2	*4.0*	6.6	*4.5*	0.103	0.040
Sweet & snacks (g/1000 kcal)	13.4	*13.9*	12.5	*13.2*	11.6	*12.7*	11.0	*14.0*	9.8	*13.5*	0.201	0.531
Fats & Oils (g/1000 kcal)	9.7	*5.1*	8.9	*4.3*	8.5	*4.2*	8.1	*4.1*	7.4	*4.0*	<0.001	<0.001
Soy beans and product (g/1000 kcal)	25.9	*14.5*	34.7	*18.0*	39.7	*19.7*	44.6	*22.1*	53.3	*28.6*	<0.001	<0.001
Fruit (g/1000 kcal)	72.2	*52.5*	78.5	*56.1*	82.3	*56.2*	86.7	*57.0*	92.8	*62.4*	<0.001	<0.001
Green & yellow vegetable (g/1000 kcal)	28.9	*16.8*	38.3	*20.7*	45.1	*21.9*	54.0	*26.0*	70.2	*35.2*	<0.001	<0.001
Other vegetable (g/1000 kcal)	77.7	*32.7*	87.8	*33.5*	97.2	*36.9*	99.9	*39.8*	108.3	*43.2*	<0.001	<0.001
Mushrooms (g/1000 kcal)	4.5	*6.3*	5.4	*6.2*	6.5	*7.0*	6.4	*6.8*	8.1	*9.2*	<0.001	<0.001
Sea algae (g/1000 kcal)	2.3	*2.7*	3.1	*3.7*	3.5	*3.8*	4.4	*4.8*	6.4	*7.5*	<0.001	<0.001
Condiment & beverage (g/1000 kcal)	52.6	*50.7*	46.9	*39.2*	43.1	*38.5*	41.9	*37.0*	39.3	*33.4*	<0.001	<0.001
Fish & shellfish (g/1000 kcal)	42.8	*19.5*	50.2	*21.4*	52.7	*22.2*	57.3	*23.5*	61.5	*25.0*	<0.001	<0.001
Meat (g/1000 kcal)	28.8	*15.3*	29.0	*15.1*	29.4	*15.0*	29.4	*15.5*	29.9	*17.5*	0.336	0.137
Egg (g/1000 kcal)	18.8	*9.4*	20.7	*10.4*	21.3	*10.3*	22.1	*10.4*	22.5	*11.4*	<0.001	<0.001
Milk & dairy (g/1000 kcal)	58.5	*43.9*	57.9	*45.5*	58.5	*44.0*	60.5	*45.5*	62.2	*50.0*	<0.001	0.001
Other food (g/1000 kcal)	2.6	*3.9*	2.2	*3.6*	2.1	*3.7*	2.2	*3.8*	1.7	*3.4*	0.061	0.268

*P* diff values obtained by ANOVA or Chi square statistics. *P* trend obtained by contrast statement of analysis of variance.

**Table 8.** Nutrient intakes of different food group according to quintiles of dietary iron intake for women: NIPPON DATA90

	Iron intake quintiles (mg/1000 kcal)											
	
Range	2.0–5.1		5.1–5.6		5.6–6.2		6.2–6.9		6.9–17.5		*P* diff	*P* trend
	
	Mean	*SD*	Mean	*SD*	Mean	*SD*	Mean	*SD*	Mean	*SD*		
*n*	970		971		970		971		972			
Cereals (g/1000 kcal)	149.5	*27.4*	144.4	*27.0*	142.3	*25.7*	138.1	*31.0*	133.5	*28.7*	<0.001	<0.001
Rice (g/1000 kcal)	100.6	*30.4*	99.8	*29.3*	98.5	*28.3*	97.7	*31.7*	93.2	*28.5*	<0.001	0.018
Flour product (g/1000 kcal)	46.7	*27.9*	42.9	*26.2*	42.3	*28.0*	39.2	*25.3*	38.3	*27.1*	<0.001	<0.001
Nuts (g/1000 kcal)	0.6	*1.9*	0.7	*2.1*	0.8	*2.3*	0.9	*2.2*	1.1	*2.4*	<0.001	<0.001
Potatoes (g/1000 kcal)	29.4	*19.6*	33.5	*21.5*	35.5	*21.3*	38.4	*23.4*	41.4	*24.7*	<0.001	<0.001
Sugar & sweetener (g/1000 kcal)	5.9	*4.2*	6.3	*5.0*	6.4	*4.8*	6.2	*3.9*	6.6	*4.5*	0.005	0.128
Sweet & snacks (g/1000 kcal)	13.4	*13.9*	12.5	*13.2*	11.6	*12.7*	11.0	*14.0*	9.8	*13.5*	<0.001	<0.001
Fats & Oils (g/1000 kcal)	9.7	*5.1*	8.9	*4.3*	8.5	*4.2*	8.1	*4.1*	7.4	*4.0*	<0.001	<0.001
Soy beans and product (g/1000 kcal)	25.9	*14.5*	34.7	*18.0*	39.7	*19.7*	44.6	*22.1*	53.3	*28.6*	<0.001	<0.001
Fruit (g/1000 kcal)	72.2	*52.5*	78.5	*56.1*	82.3	*56.2*	86.7	*57.0*	92.8	*62.4*	<0.001	<0.001
Green & yellow vegetable (g/1000 kcal)	28.9	*16.8*	38.3	*20.7*	45.1	*21.9*	54.0	*26.0*	70.2	*35.2*	<0.001	<0.001
Other vegetable (g/1000 kcal)	77.7	*32.7*	87.8	*33.5*	97.2	*36.9*	99.9	*39.8*	108.3	*43.2*	<0.001	<0.001
Mushrooms (g/1000 kcal)	4.5	*6.3*	5.4	*6.2*	6.5	*7.0*	6.5	*6.8*	8.1	*9.6*	<0.001	<0.001
Sea algae (g/1000 kcal)	2.3	*2.7*	3.1	*3.7*	3.5	*3.8*	4.4	*4.8*	6.4	*7.5*	<0.001	<0.001
Condiment & beverage (g/1000 kcal)	52.6	*50.7*	46.9	*39.2*	43.1	*38.5*	41.9	*37.0*	39.3	*33.4*	<0.001	<0.001
Fish & shellfish (g/1000 kcal)	42.8	*19.5*	50.2	*21.4*	52.7	*22.2*	57.3	*23.5*	61.5	*25.0*	<0.001	<0.001
Meat (g/1000 kcal)	28.8	*15.3*	29.0	*15.1*	29.4	*15.0*	29.4	*15.5*	29.8	*17.5*	0.599	0.332
Egg (g/1000 kcal)	18.8	*9.4*	20.7	*10.4*	21.3	*10.3*	22.1	*10.4*	22.5	*11.4*	<0.001	<0.001
Milk & dairy (g/1000 kcal)	58.5	*43.9*	57.9	*45.5*	58.5	*44.0*	60.2	*45.5*	62.2	*50.0*	0.237	0.404
Other food (g/1000 kcal)	2.6	*3.9*	2.2	*3.6*	2.1	*3.7*	2.2	*3.8*	1.7	*3.4*	<0.001	0.009

*P* diff values obtained by ANOVA or Chi square statistics. *P* trend obtained by contrast statement of analysis of variance.

Table 9 shows the iron and major food group intake for men and women by age-groups in cohorts of NIPPON DATA80 and 90, respectively. The dietary iron intake and major food group intakes were significantly associated with age (all *P* < 0.001). Similar was also observed for the men and women participants of NIPPON DATA90 (Table 10).

**Table 9.** Dietary intake of iron and other major nutrient by age group in men and women for NIPPON DATA80

Age (years)	30–39		40–49		50–59		60–69		70–		Trend *P*	*P* diff

Variable	Mean	SD	Mean	SD	Mean	SD	Mean	SD	Mean	SD		
Men (*n*)	1220		1196		1019		679		471			
Iron (mg/1000 kcal)	6.0	*1.1*	6.1	*1.1*	6.3	*1.1*	6.8	*1.3*	6.7	*1.3*	<0.001	<0.001
Iron (mg/day)	14.9	*3.9*	15.2	*3.8*	15.8	*3.9*	15.4	*4.0*	13.2	*3.5*	0.003	<0.001
Carbohydrate (%kcal)	57.8	*5.8*	58.6	*5.8*	59.7	*6.2*	62.0	*6.5*	64.5	*6.7*	<0.001	<0.001
Protein (%kcal)	14.7	*2.1*	15.0	*2.0*	15.3	*2.2*	15.3	*2.1*	15.1	*2.2*	<0.001	<0.001
Total fat (%kcal)	22.1	*5.1*	20.4	*4.7*	19.4	*5.0*	18.3	*5.0*	17.2	*4.9*	<0.001	<0.001
Total energy (kcal)	2475.2	*486.0*	2476.2	*450.0*	2487.7	*484.2*	2297.8	*541.4*	1981.5	*406.4*	<0.001	<0.001
Women (*n*)	1583		1469		1319		900		566			
Iron (mg/1000 kcal)	6.5	*1.1*	6.9	*1.2*	7.4	*1.4*	7.3	*1.4*	7.4	*1.4*	<0.001	<0.001
Iron (mg/day)	12.8	*3.0*	13.9	*3.6*	14.5	*3.7*	13.2	*3.8*	12.1	*3.2*	<0.001	<0.001
Carbohydrate (%kcal)	59.2	*5.8*	60.9	*6.2*	62.6	*6.5*	65.5	*6.8*	66.4	*7.0*	<0.001	<0.001
Protein (%kcal)	15.1	*1.9*	15.3	*2.1*	15.7	*2.3*	15.7	*2.2*	15.7	*2.3*	<0.001	<0.001
Total fat (%kcal)	23.9	*5.4*	22.8	*5.6*	21.2	*5.4*	18.8	*5.4*	18.5	*5.2*	<0.001	<0.001
Total energy (kcal)	1957.0	*338.5*	2025.5	*400.6*	1984.9	*420.4*	1825.4	*412.8*	1639.2	*341.7*	<0.001	<0.001

Values are in mean and *standard deviation*. Trend *P* obtained by contrast statement of analysis of variance. *P* diff values obtained by ANOVA statistics.

**Table 10.** Dietary intake of iron and other major nutrient by age group in men and women for NIPPON DATA90

Age (years)	30–39		40–49		50–59		60–69		70–		Trend *P*	*P* diff

Variable	Mean	SD	Mean	SD	Mean	SD	Mean	SD	Mean	SD		
Men (*n*)	660		836		793		708		491			
Iron (mg/1000 kcal)	5.1	*0.9*	5.4	*1.0*	5.7	*1.2*	5.7	*1.2*	5.9	*1.3*	<0.001	<0.001
Iron (mg/day)	12.1	*2.8*	12.8	*3.3*	13.9	*3.7*	12.7	*3.6*	11.6	*3.3*	<0.001	<0.001
Carbohydrate (%kcal)	55.2	*5.1*	54.9	*4.9*	56.2	*5.5*	58.3	*5.8*	60.5	*6.2*	<0.001	<0.001
Protein (%kcal)	15.0	*1.7*	15.6	*1.9*	15.9	*2.0*	15.6	*1.9*	15.7	*2.1*	<0.001	<0.001
Total fat (%kcal)	24.6	*4.2*	23.2	*3.9*	21.9	*4.2*	21.1	*4.6*	20.3	*4.5*	<0.001	<0.001
Total energy (kcal)	2376.7	*436.1*	2405.1	*427.2*	2446.2	*475.3*	2237.8	*414.0*	1984.7	*413.5*	<0.001	<0.001
Women (*n*)	1031		1171		1035		915		702			
Iron (mg/1000 kcal)	5.5	*0.9*	5.9	*1.1*	6.3	*1.4*	6.4	*1.3*	6.3	*1.3*	<0.001	<0.001
Iron (mg/day)	10.2	*2.3*	11.5	*2.9*	12.2	*3.5*	11.5	*3.1*	10.1	*2.7*	<0.001	<0.001
Carbohydrate (%kcal)	55.8	*4.9*	56.9	*5.1*	59.4	*5.8*	61.3	*6.3*	62.7	*6.0*	<0.001	<0.001
Protein (%kcal)	15.3	*1.7*	16.0	*1.9*	16.3	*2.0*	16.1	*2.1*	16.0	*2.1*	<0.001	<0.001
Total fat (%kcal)	27.3	*4.4*	25.8	*4.2*	23.9	*4.7*	22.4	*4.8*	21.2	*4.7*	<0.001	<0.001
Total energy (kcal)	1880.2	*313.9*	1964.7	*350.4*	1926.5	*371.1*	1808.9	*374.7*	1615.5	*325.8*	<0.001	<0.001

Values are in mean and *standard deviation*. Trend *P* obtained by contrast statement of analysis of variance. *P* diff values obtained by ANOVA statistics.

## DISCUSSIONS

This cross-sectional study was initiated to describe the dietary iron intake and its relation with other dietary factors and clinical characteristics among a representative sample cohort of Japanese population. We found significant correlation between the age and the dietary iron intake. For both men and women older aged people had higher iron intake. We also observed significant association for blood pressure indices, which understandably reflected the correlation observed for age. Dietary protein was associated with iron intake where as fat and carbohydrate did not show any association. Regarding the minerals, significant correlation was observed between the different minerals and dietary iron intake. These dietary patterns had not been highlighted adequately in prior nutrition surveys, and should be confirmed by additional analysis of NIPPON DATA and NNS or other studies. Our findings are potentially useful for the development of hypothesis, future NIPPON DATA studies to examine and interpret interactions in detail between iron intake and other nutritional factors, food types, risk factors and mortality from various cardiovascular outcomes.

Among the nutrients, iron is an essential mineral. Iron status, measured as dietary iron intake, total-iron-binding capacity, ferritin, transferrin saturation, and total body iron have been evaluated as potential markers of cardiovascular disease in human epidemiologic research.^10^^–^^12^ High levels of iron stores over time, especially heme-iron from animal sources, have been implicated in the possible pathogenesis of several common diseases of aging including malignancy, neurodegenerative disorders, diabetes mellitus, infections, and atherosclerosis.^13^^–^^17^ Iron deficiency has been related with impaired neuropsychological function, reduced worker productivity, lowered immunity and decreased metabolic rate.^18^^–^^20^ Furthermore, low dietary iron availability is a major cause of anemia and anemia is an independent risk factor for CVD outcomes.^21^ But simultaneously the genetic, other pathological and environmental factors may also contribute to iron-related CVD risk.^22^

Dietary iron intake is likely to be associated with the subject level covariates as well as the other components of the dietary patterns of a person. Uniquely among developed countries, dietary habits rendered Japanese populations to their relatively low intake of iron and high intake of iron absorption inhibitors.^23^ Distinctive feature of Japanese dietary pattern related to iron intake comprise lower meat consumption, which results in lower total iron intake, particularly lower haem iron intake.^23^ Simultaneously Japanese diet has abundance for the intake of higher amounts and a wider range of inhibitors of iron absorption, such as soyabeans and green tea, which are traditional Japanese foods.^23^^–^^26^ But, the Japanese have changed their lifestyle as well as dietary habits over the past years,^27^^,^^28^ which have shifted from ‘traditional’ to ‘westernized’ diet. The dangers of this dietary and life style shift include the emerging problems of obesity, hyperlipidemia, and diabetes mellitus making the overall CVD risk factor scenario unfavorable.^29^^,^^30^ Reflecting the major change in the dietary habit, the recent trends in food intake by food groups have shown a decreasing consumption of rice, fish and shellfish, soybeans and soybean products. On the other hand, increasing consumption of meat and poultry, milk and dairy products have also been observed. In our study we observed these consumption patterns to be associated with higher dietary iron, which reflects the worsening of the risk-scenario of CVD. We also found that the participants with higher intake of dietary iron tended to be from older age categories. These findings are comparable to the National Nutrition Survey in 2003 findings,^31^ which demonstrated that iron intake of people aged 50 and over was higher than younger people. The National Nutrition Survey in 2003 was based on the individual nutrition intake data.^31^ Regarding the association between age and dietary iron intake, similar to our findings, Iso et al^32^ also reported lower intake of iron among people aged 40 to 49 years using FFQ.

In our study population we were not able to identify if some were on iron supplementation. Additionally we also did not have information regarding the post-menopausal status of the women participants of menopausal age-groups. In regards to the comparison of dietary iron intake between NIPPON DATA80 and 90, the observed lower iron intake in the 90 cohort should be attributed to the difference in the estimation between the two cohorts.^5^ For NNS-80, 3rd revised edition^33^ of the standard tables of food composition in Japan was used, and the 4th edition^34^ was used for the NNS-90. The lower iron intake estimated from the representative nutrient composition seemed to be related to the corresponding food table used to calculate nutrient intakes.^5^

The recent epidemiological studies reported that that the previously reported declining trend in stroke may have leveled off or slowed down greatly^35^ and the recent ischemic heart disease trend might have an increasing pattern in Japanese population.^36^^,^^37^ In the current backdrop, information about the iron intake in Japanese population in relation to other nutrient intake and overall dietary habit may elucidate information which might be used for primary prevention of CVD, and the effect of the dietary iron intake needs to be further investigated using longitudinal research for crafting effective public health intervention against this probable risk.
